# Tumor-Stroma Interaction in PDAC as a New Approach for Liquid Biopsy and its Potential Clinical Implications

**DOI:** 10.3389/fcell.2022.918795

**Published:** 2022-05-26

**Authors:** Julian Götze, Christine Nitschke, Faik G. Uzunoglu, Klaus Pantel, Marianne Sinn, Harriet Wikman

**Affiliations:** ^1^ Department of Oncology, Hematology and Bone Marrow Transplantation with Section Pneumology, University Cancer Center Hamburg, Hamburg, Germany; ^2^ Institute of Tumor Biology, University Medical Center Hamburg-Eppendorf, Hamburg, Germany; ^3^ Department of General, Visceral and Thoracic Surgery, University Medical Center Hamburg-Eppendorf, Hamburg, Germany

**Keywords:** pancreatic cancer, liquid biopsy, cancer-associated fibroblasts (CAFs), tumor microenvironment, circulating tumor cells (CTCs)

## Abstract

The extremely poor prognosis for patients with pancreatic ductal adenocarcinoma (PDAC) has remained unchanged for decades. As a hallmark of PDAC histology, the distinct desmoplastic response in the tumor microenvironment is considered a key factor exerting pro- and antitumor effects. Increasing emphasis has been placed on cancer-associated fibroblasts (CAFs), whose heterogeneity and functional diversity is reflected in the numerous subtypes. The myofibroblastic CAFs (myCAFs), inflammatory CAFs (iCAFs) and antigen presenting CAFs (apCAFs) are functionally divergent CAF subtypes with tumor promoting as well as repressing effects. Precise knowledge of the underlying interactions is the basis for a variety of treatment approaches, which are subsumed under the term antistromal therapy. Clinical implementation is still pending due to the lack of benefit—as well as paradoxical preclinical findings. While the prominent significance of CAFs in the immediate environment of the tumor is becoming clear, less is known about the circulating (c)CAFs. cCAFs are of particular interest as they seem not only to be potential new liquid biopsy biomarkers but also to support the survival of circulating tumor cells (CTC) in the bloodstream. In PDAC, CTCs correlate with an unfavorable outcome and can also be employed to monitor treatment response, but the current clinical relevance is limited. In this review, we discuss CTCs, cCAFs, secretomes that include EVs or fragments of collagen turnover as liquid biopsy biomarkers, and clinical approaches to target tumor stroma in PDAC.

## 1 Introduction

Pancreatic ductal adenocarcinoma (PDAC) is one of the most lethal cancers with a 5-year survival rate of <9% over all stages and remains a major challenge for current cancer therapy (Rawla et al., 2019). In the Western world PDAC is predicted to become the second leading cause of cancer-associated death (Malvezzi et al., 2019; Sung et al., 2021). More than 80% of cases are diagnosed at an advanced or metastatic stage, hence most treatment combination regimen rely on chemotherapy (Kuehn, 2020; Wang et al., 2021). A dense fibroblastic stroma is a hallmark of the PDAC microenvironment, which can constitutes up to 90% of the tumor mass (Ansari et al., 2017). The PDAC associated stroma mainly consists of extracellular matrix (ECM) proteins and cancer-associated fibroblasts (CAFs) and functions as a barrier of effective drug penetration (Jacobetz et al., 2013). Furthermore, immune cells, e.g., myeloid cells and lymphocytes, and to a lower extent angiogenic vascular cells are present (Hanahan and Coussens, 2012; Chen and Mellman, 2017; Ren et al., 2018). Considered as critical players in PDAC carcinogenesis CAFs are engaged in a dynamic exchange with cancer cells based on a variety of mechanisms ranging from paracrine secretion to extracellular vesicle (EV) trafficking (von Ahrens et al., 2017; Leca et al., 2016). Given that not only tumor promoting but also tumor repressive effects are mitigated by the tumor-stromal crosstalk, the CAFs in the center are of paramount interest with respect to novel therapeutic approaches (Wang et al., 2021).

The current unmet need for clinically relevant prognostic and predictive biomarkers is considered another reason for the persistently poor prognosis of PDAC patients (Wang et al., 2021). Liquid biopsy has become a powerful non-invasive tool to obtain important tumor related information from body fluids. The number of CTCs correlate with a poor prognosis and can also be used to monitor therapy response (Alix-Panabières and Pantel, 2021). However, the clinical impact of CTC enumeration regarding survival is limited (Ferreira et al., 2016). Meanwhile, the detection of circulating (c)CAFs, as surrogates of the tumor-associated stroma, is a merging novel field in liquid biopsies (Ortiz-Otero et al., 2020a).

In view of their potential use as future liquid biopsy markers and as clinical approaches for tumor stroma targeting, this review summarizes the current knowledge about CTCs, cCAFs and their stroma-derived proteomic signature.

## 2 CAFs as the Predominant Cell Type of the Tumor Microenvironment

### 2.1 Characteristics of CAFs

Today, it is well accepted, that CAFs are composed of a large number of cells from different origins—leading to great heterogeneity in terms of phenotype and function, which hinders a consistent classification. Therefore, the key features were summarized in a consensus statement: elongated cells that are negative for lineage-specific epithelial, endothelial, and leukocyte markers and do not harbor mutations of their associated tumor are referred to as CAFs (Sahai et al., 2020). However, the lack of a specific marker compromises accurate tracing of their cellular origin (Nurmik et al., 2020).

### 2.2 Origin of CAFs as a Source of Heterogeneity and Subpopulations

Originally, CAFs in PDAC were considered to be derived from tissue-derived fibroblasts and in a majority from pancreatic stellate cells (PSCs) (Erkan et al., 2012; Arina et al., 2016). This paradigm has been challenged by recent findings on the lineage of CAFs. After targeted ablation of PSCs in a PDAC mouse model, it was observed that only a minority of PSCs contribute to the population of total CAFs (Helms et al., 2022). PSCs are mesenchymal tissue-resident cells characterized by vitamin A-containing lipid droplet storage function and expression of fibroblast activation protein *α* (FAP*α*) (Erkan et al., 2012; Moir et al., 2015). FAP*α* exerts proteolytic activity and is linked to ECM degradation and remodeling (Knopf et al., 2015). Upon paracrine stimulation from adjacent tumor cells, quiescent PSCs become activated, then termed CAFs, and increase the production of ECM proteins (Bachem et al., 2005).

**FIGURE 1:**
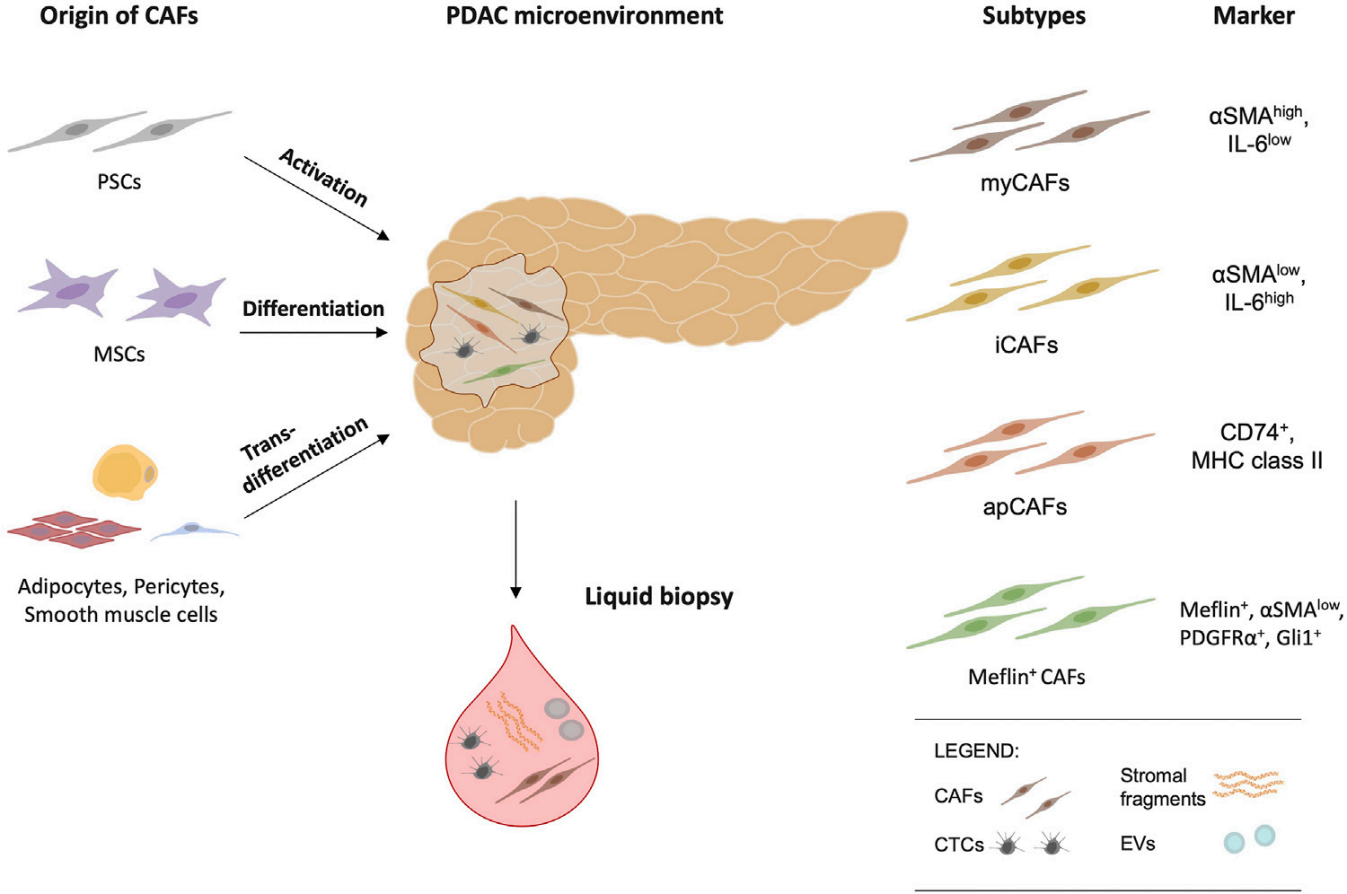
Cancer-associated fibroblasts (CAFs) have multiple origins and arise from pancreatic stellate cells (PSCs), mesenchymal stem cells (MSCs), adipocytes, pericytes, and smooth muscle cells. Myofibroblastic CAFs (myCAFs), inflammatory CAFs (iCAFs), antigen-presenting CAFs (apCAFs) and Meflin-positive CAFs are the major CAF subtypes and reflect functional diversity. As potential biomarkers, circulating tumor cells (CTCs), circulating (c)CAFs, stromal fragments, and extracellular vesicles (EVs) are an emerging area of research in liquid biopsy and offer future clinical implications.

However, as part of a versatile multicellular origin, transdifferentiation of epithelial and endothelial cells (Iwano et al., 2002; Zeisberg et al., 2007), as well as adipocytes, pericytes and smooth muscle cells can give rise to CAFs (Dulauroy et al., 2012; Bochet et al., 2013). Moreover, a transgenic mouse model indicated that a relevant subset of CAFs appears to be additionally recruited from bone marrow niche cells and in fact arise from mesenchymal stem cells (MSCs) (Quante et al., 2011) (Figure 1).

The different subtypes appear to have partially contrasting effects on tumor biology, so accurate knowledge of these subpopulations is crucial for understanding and developing therapeutic approaches.

### 2.3 CAF Subtypes

This section highlights shortly the most important CAF subtypes currently described. However, numerous other CAF subtypes have been identified in recent years. Summarizing them all is beyond the scope of this overview—yet they have recently been described and reviewed (Awaji and Singh, 2019; Neuzillet et al., 2019; Pereira et al., 2019; Garcia et al., 2020; Helms et al., 2020; Huang and Brekken, 2020).

Single cell resolution studies, including single-cell RNA sequencing, have provided valuable insights into the composition and properties of stromal cells (Öhlund et al., 2017; Elyada et al., 2019; Biffi et al., 2019). In a 3D-coculture system of PSCs and PDAC cells Öhlund et al. described two distinct subtypes, myofibroblastic CAFs (myCAFs) and inflammatory CAFs (iCAFs), thereby confirming their functional heterogeneity (Öhlund et al., 2017) (Figure 1). In addition, these subtypes seem to differ in their spatial distribution. MyCAFs are FAP*α*-positive cells with high expression of *α*-smooth muscle actin (*α*SMA) and are in direct contact with cancer cells (Öhlund et al., 2017). Activated by the proximity to cancer cells, myCAFs increase their collagen I synthesis (Öhlund et al., 2017). However, counterintuitively, depletion of *α*SMA-positive CAFs impaired survival in mice despite reduction in total collagen content (Özdemir et al., 2014). Thus, these results imply a possible antitumor activity of myCAFs (Özdemir et al., 2014).

The iCAF subtype is located more distant from tumor cells and showed an upregulation of leukemia inhibitory factor (LIF), C-X-C Motif Chemokine Ligand 1 (CXCL1), and Interleukin 6 (IL-6) and IL-11 (Öhlund et al., 2017). In a PDAC mouse model, inhibition of LIF and genetic depletion of its receptor resulted in increased sensitivity to gemcitabine treatment and impaired tumor growth (Shi et al., 2019). Further studies have identified additional markers for iCAFs including CXCL12, IL6, and IL8 as wells as TNF*α* and NF-κB (Elyada et al., 2019). By secreting pro-inflammatory cytokines and mediating immune evasion, iCAFs are widely regarded as promoters of tumor growth in PDAC (Biffi et al., 2019).

Previous work has illustrated that myCAFs and iCAFs can switch subtypes, thereby indicating a high level of plasticity. These subpopulations are therefore conceptualized as functional states on a continuum rather than endpoints of differentiation. Through JAK/STAT activation, IL-1 promotes the development of inflammatory CAFs. Whereas TGF*β* counteracts the JAK/STAT signaling cascade by suppressing expression of Il1r1, thereby mediating a transition to the myCAF type (Biffi et al., 2019). Moreover, inhibition of the TGF*β* pathway resulted in elevated expression of iCAF marker genes (Biffi et al., 2019).

Recently scRNA-seq of tumors in a PDAC mouse model revealed a novel CAF subtype that expresses MHC class II family genes (Elyada et al., 2019). The upregulated signaling pathways and genes, e.g., histocompatibility 2, class II antigen A (H2-Aa) and H2-Ab1, are typically associated with antigen-presenting cells (APCs), hence this subtype was referred to as antigen-presenting CAFs (apCAFs) (Elyada et al., 2019).

Conceptually, another classification proposal for this complex, growing landscape of different CAF subtypes is to divide CAFs into cancer-promoting CAFs (pCAFs) and cancer-restraining CAFs (rCAFs) (Kalluri, 2016; Kobayashi et al., 2019; Mizutani et al., 2019). However, the definition of pCAFs and rCAFs still needs to be clarified (Kalluri, 2016). Inclusion of the subgroups described above, such as iCAFs, apCAFs, and myCAFs, in this classification is also challenging. This is partly due to the multiple functions of their markers, exemplified by the controversial role of alpha-SMA in the progression of PDAC (Özdemir et al., 2014). Mizutani et al. discovered a potential marker for rCAFs, namely meflin, a glycosylphosphatidyl inositol (GPI)-anchored protein specifically expressed in MSCs (Mizutani et al., 2019). The Meflin-positive CAF subtype is defined by its positivity for Meflin, Platelet-derived growth factor receptor-*α* (PDGFR*α*), Glioma-associated oncogene homologue 1 (Gli1) with low expression of *α*SMA (Mizutani et al., 2019). The infiltration of Meflin-positive CAFs in human PDAC tissue samples appears to have prognostic value as it was correlated with beneficial outcome (Mizutani et al., 2019). This observation was also supported in a meflin-deficient PDAC mouse model. Thus, ablation of meflin promoted tumor growth along with a poorly differentiated histology (Mizutani et al., 2019).

Knowledge of CAF subtypes in PDAC is clearly increasing, but their precise implications for treatment response and overall survival (OS) has yet to be determined.

## 3 Detection of CTCs and CAFs in Liquid Biopsy

Liquid biopsy has become a powerful non-invasive tool to obtain important tumor related information from body fluids. Most clinically relevant data has been collected from the analyses of circulating tumor DNA (ctDNA) and circulating tumor cells (CTCs). The presence of CTCs in the bloodstream of cancer patients not only correlate with a poor prognosis but can also be used to monitor the therapy response and collect tumor material for molecular analyses (Alix-Panabières and Pantel, 2021). Whereas numerous studies on the biological and clinical role of CTC in PDAC exist today, the detection of cCAFs is a merging novel field (Ao et al., 2015; Ortiz-Otero et al., 2020a).

### 3.1 Current Value of the CTCs in PDAC

CTCs in the blood of cancer patients are a rare subset of the most malignant tumor cells, representing the whole tumor burden (Yeo et al., 2022). In non-resectable PDAC patients, the gain of tumor tissue is often difficult. CTCs might therefore become an important alternative tool due to its potential utility as diagnostic, prognostic, and predictive biomarker (Pantel and Alix-Panabières, 2019; Yeo et al., 2022). Circulating epithelial cells have been found in pancreatic precancerous lesions such as intraductal papillary mucinous neoplasm (IPMN) indicative of highly migratory capabilities of pancreatic cells even in early tumor setting (Franses et al., 2018).

There is no gold-standard CTC detection enrichment method yet for PDAC, and low detection rates, small cohorts and a focus on locally advanced and metastatic stages hinder valuable comparisons between the existing studies that have assessed CTC detection in peripheral blood of PDAC patients (Gall et al., 2019; Lee et al., 2019; Yeo et al., 2022). Previous studies have included between 14 and 172 PDAC patients each, with reported CTC rates between 7 and 42% for operable patients and 19 and 48% for metastatic patients (Ferreira et al., 2016; Effenberger et al., 2018; Martini et al., 2019; Yeo et al., 2022). Many of these studies have proven that CTCs are independent predictors of bad prognosis and could facilitate a better stratification of patients than classical parameters (TNM classification, imaging methods, CA-19-9 levels) (Ferreira et al., 2016; Effenberger et al., 2018; Martini et al., 2019). Two meta analyses, comprising more than 600 patients each, have proved the association of CTC positivity and worse survival (Han et al., 2014; Ma et al., 2014).

Due to the large heterogeneity seen in PDAC CTCs, physical enrichment techniques combined with optimized CTC detection seem promising (Kalluri and Neilson, 2003; Kalluri and Weinberg, 2009; Martini et al., 2019). Another promising approach for increasing CTC detection rates is to analyze the tumor-draining blood—as CTCs are released directly into the tumor-draining portal vein before entering the liver as a first capillary bed (Arnoletti et al., 2017; Song et al., 2020; White et al., 2021; Arnoletti et al., 2022).

CTCs can be found as single cells or cell clusters (multicellular CTC aggregates called CTC clusters). The CTC cluster can be composed of only CTCs (homotypic clusters) or admixed of CTCs and immune or stromal cells (heterotypic clusters) (Aceto, 2020; Wrenn et al., 2021). Although CTC clusters are much more rarely seen than single CTCs, their presence has been associated with higher metastatic potential in animal models (Cheung et al., 2016; Liu et al., 2019; Taftaf et al., 2021) and indicated worse prognosis than single CTCs (Hou et al., 2012; Aceto et al., 2014). Also, among PDAC patients, clusters have been identified (Wu et al., 2018). The heterotypic clusters can be composed of fibroblasts, endothelial cells, white blood cells, or platelets (Aceto et al., 2014). In a lung cancer mouse model, the viability of CTC was higher if they formed heterotypic clusters with fibroblasts (Duda et al., 2010). Importantly, depletion of CAFs resulted in reduced metastasis, implying their essential role in CTC survival and metastatic capacity (Duda et al., 2010).

In future personalized-medicine approaches, characterization and functional testing of CTCs and clusters might lead to benefits for patient management and uncover new therapies by identifying the specific CTC subpopulations for metastatic progression (Yeo et al., 2022).

### 3.2 State of Knowledge on Detection of cCAFs Using Liquid Biopsy Approaches in Cancer and Potential Application in PDAC

In various tumor entities including PDAC, cCAFs have been detected (Ortiz-Otero et al., 2020a). These rare cells can be found in a heterotypic cluster with CTCs or alone (Ao et al., 2015; Sharma et al., 2021). In general, patients at a metastatic stage showed a significantly higher amount of cCAFs in the blood compared to patients with localized tumors (Ao et al., 2015). In a cohort of 34 patients with metastatic breast cancer, cCAFs were found in 88.2%, with numbers varying notably, ranging from 0 to 117 per 7.5 ml of EDTA blood. In contrast, cCAFs were present in only 23.1% of the 13 patients with localized cancer, and the maximum count per 7.5 ml of blood did not exceed 2 (Ao et al., 2015). The potential use of cCAFs as a predictive biomarker is also supported by the observation that cCAFs were not present in healthy donors and an increased cCAF count correlated with poor prognosis (Ao et al., 2015; Ortiz-Otero et al., 2020a).

Previous studies have employed different enrichment methods and surface markers to detect cCAFs, thereby limiting their comparability and predictions on the occurrence of cCAFs in different tumor types (Ao et al., 2015; Ortiz-Otero et al., 2020a). Ao et al. first exploited the physical properties of cCAFs by applying a size-based microfilter to enrich cCAFs from peripheral blood samples of patients with breast cancer (Ao et al., 2015). Subsequently, the classification of cCAFs was conducted by the combined detection of FAP*α* and *α*SMA. As an additional morphological condition, the spindle-shaped form of the captured cells, a feature typical of CAFs, was verified (Ao et al., 2015). Another study analyzed the blood of patients with prostate cancer using the CellSearch^TM^ system. Fibroblast-like cells, defined as CK- and CD45-negative and vimentin-positive populations, were observed in patients with metastatic but not localized disease stage in this study (Jones et al., 2013). High expression of vimentin is indeed found on fibroblasts. Nevertheless it should be taken into account that other cells of mesenchymal origin and tumor cells undergoing EMT also express vimentin (Kalluri and Weinberg, 2009).

The studies by Ortiz-Ortero et al. covered the most extensive variety of different tumor types, including six patients with metastatic PDAC (Ortiz-Otero et al., 2020a). In their approach, mononuclear cells were isolated using Ficoll density centrifugation, followed by positive enrichment based on anti-fibroblast magnetic beads and identification by *α*SMA staining (Ortiz-Otero et al., 2020a).

As local CAFs are of paramount importance in the tumor microenvironment (Sahai et al., 2020), it is reasonable to speculate that cCAFs alone or in a heterotypic cluster with CTCs might also have analogous biological significance, e.g., facilitating metastasis (Ortiz-Otero et al., 2020b). This remains a hypothesis, as there is much less known about cCAFs than CTCs, although initial data are promising.

### 3.3 Stroma-Derived Proteomic Signature Measured in the Bloodstream as a Biomarker in PDAC

Since cCAF are rare and a heterogeneous population of cells, a combined detection approach including CTCs might be beneficial to increase sensitivity. Furthermore, it is conceivable to exploit other PDAC specific stromal characteristics: ECM proteins as hyaluronan (HA) along with collagen types I, III, and IV, are surrogates for desmoplasmic expanse in PDAC (Whatcott et al., 2015). This abundant accumulation of ECM caused by the increased activity of CAFs is also subject to enzyme-mediated turnover, e.g., by matrix metalloproteinases, the fragments of which may, in turn, be released into the bloodstream. Several studies have explored the value of these blood-detectable stromal fragments as potential biomarkers in PDAC (Willumsen et al., 2013; Willumsen et al., 2019; Chen et al., 2020; Nissen et al., 2021). In a cohort of 972 participants, including 809 patients with PDAC, serum levels of HA and the N-terminal propeptide of type III collagen (PRO-C3) were measured and compared with healthy subjects. Levels of HA and PRO-C3 were significantly elevated in patients with PDAC compared to healthy subjects or patients with benign diseases, including IPMN (Chen et al., 2020). Another study demonstrated the association between a higher PRO-C3 value and worse OS. Moreover, the higher ratio of fragment C3M, the MMP-mediated degradation product of type III collagen, to PRO-C3 was associated with longer OS and can be interpreted as a sign of a decreased fibrotic activity (Willumsen et al., 2019).

However, the crosstalk of CAFs and PDAC cancer cells occurs not only by paracrine mechanisms but also through EV trafficking. When CAFs are exposed to unfavorable pathophysiological conditions, such as lipid deficiency, macrophage co-cultivation and hypoxia, they secrete EVs bearing the complex annexin A6/LDL receptor-related protein 1/thrombospondin 1 (ANXA6/LRP1/TSP1) (Leca et al., 2016). Uptake of this complex by cancer cells resulted in increased tumor aggressiveness. Interestingly, Leca et al. detected CAF-derived ANXA6-positive EVs exclusively in the serum of PDAC patients compared with healthy subjects and subsequently showed that their occurrence was linked to shorter OS (Leca et al., 2016).

In the future, information on the fibrotic activity or aggressiveness of PDAC obtained by liquid biopsy could be an effective tool for PDAC subclassification and treatment stratification. Blood-based measurements of stromal fragments or CAF-derived EVs could be a valuable contribution to this purpose.

## 4 Addressing Desmoplasia—A New Approach for Therapeutic Options

Stromal and CAF depletion as a therapeutic concept in PDAC is an intense area of research with numerous ongoing preclinical and translational studies (Jiang et al., 2020; Geng et al., 2021). From the plethora of potentially actionable pathways and targets, a few have been highlighted below for their potential clinical implications in the near future.

### 4.1 Targeting the Stroma

Nab-paclitaxel was the first drug with antistromal activity to show benefit in clinically relevant studies (Von Hoff et al., 2011; Von Hoff et al., 2013). Furthermore, the HA expression is considered a negative prognostic marker in PDAC and is therefore an attractive target for drug intervention (Whatcott et al., 2015). The benefit of HA degradation by pegvorhyaluronidase-alfa has been evaluated in multiple clinical trials (Hingorani et al., 2016; Infante et al., 2018). After initially promising results, it failed to improve survival in a large phase III trial (Van Cutsem et al., 2020). Several other anti-fibrotic agents such as halofuginone have demonstrated in preclinical studies that modulation of the TME toward a lower stromal content enhances the effect of antineoplastic agents (Elahi-Gedwillo et al., 2019). However, clinical benefit has yet to be proven.

PDAC peritumoral stromal response is significantly regulated by the Sonic Hedgehog (Shh) pathway, yet Shh inhibitors have failed in clinical trials (De Jesus-Acosta et al., 2020). Inhibition of Shh led to a reduction in *α*SMA-positive CAFs, but paradoxically to more aggressive tumors, underscoring the distinct and partially protective functions of the recently identified CAF subtypes in PDAC (Rhim et al., 2014).

### 4.2 CAFs as Therapeutic Targets

Given that CAFs in part fuel tumor growth and form physical barriers impeding effective drug delivery, these cells appeared to be promising targets for therapeutic approaches (Jacobetz et al., 1136; Vonlaufen et al., 2008). An interesting target is the marker FAP*α*, which has been identified as a promoter of tumor growth, as its genetic deletion resulted in delayed tumor development and attenuation of metastasis (Lo et al., 2017). Nevertheless, inhibition of FAP*α*-positive CAFs by bispecific antibodies or chimeric antigen receptor T-cells, however, has shown no clinical value so far (Hingorani et al., 2016).

Evasion of cancer immunity and therapy resistance involves intimately also the interaction of CAFs with immune and tumor cells, which can be partially reversed by augmenting T-cell responses (Feig et al., 2013). The inhibition of CXCL12 secreted by FAP*α*-positive CAFs *via* CXCR4 antagonists increased cytotoxic T-cell infiltration and promoted an anti-*α*-PD-L1-mediated immune response in PDAC mouse models (Feig et al., 2013). In a phase II trial (NCT02826486), a CXCR-4 inhibitor in combination with PD-1-inhibition and chemotherapy led to an increase in survival in metatastic PDAC patients (Bockorny et al., 2020). Furthermore, *via* secretion of pro-inflammatory cytokines, including Il-6, iCAFs are considered a driving oncogenic force in PDAC (Geng et al., 2021). High IL-6 levels in the serum of PDAC patients are associated with cachexia and a worse prognosis (Okada et al., 1998; Rupert et al., 2021). Dual blockade of IL-6 and the immune checkpoint PD-1 acts synergistically and is hypothesized to overcome thereby immune resistance (Mace et al., 2018). The clinical utility of an IL-6 inhibitor is currently being evaluated in a combination therapy in advanced PDAC (NCT02767557).

Tumor promoting crosstalk between PDAC cancer cells and CAFs is mediated by a ligand for HER3 and HER4 receptors, neuroregulin-1, which is secreted by both cell types (Ogier et al., 2018). Its inhibition showed reduced migration and tumor growth in PDAC mouse models (Ogier et al., 2018). In an ongoing phase I/II, an anti-HER2xHER3 bispecific antibody has shown promising efficacy in neuroregulin-1 fusion-positive PDAC patients (NCT02912949).

The fact that the antifibrotic mechanisms of action and CAF heterogeneity is still rather poorly understood, exemplifies the ongoing cumbersome search for the ideal targeted therapy in PDAC.

## 5 Outlook and Possible Clinical Implementation

Within the PDAC microenvironment, CAFs predominantly imprint intrastromal cellular crosstalk and exert both tumor-promoting and tumor-suppressive effects (Wang et al., 2021). Moreover, their biological functions are as diverse as their heterogeneous origins. The more we know about this biological complexity, the more we can use partial aspects, such as blood circulation of CAFs and release of stromal fragments, for liquid biopsy approaches to establish predictive markers in PDAC (Willumsen et al., 2019; Ortiz-Otero et al., 2020a). The most promising approach for liquid biopsies in PDAC might indeed be a combined approach of CTC, cCAF and other stromal fragment detection to increase sensitivity and maximize the chance of translation liquid biopsy techniques in clinical routine with therapeutic relevance (Willumsen et al., 2019; Ortiz-Otero et al., 2020a; Yeo et al., 2022). The steps toward clinical application of antistromal therapies, in addition to nab-paclitaxel, have been marked by many setbacks (Van Cutsem et al., 2020; Geng et al., 2021). However, the numerous preclinical and ongoing clinical trials are encouraging (Geng et al., 2021).
